# Quantitative and Molecular Similarity Analyses of the Metabolites of Cold- and Hot-Natured Chinese Herbs

**DOI:** 10.1155/2021/6646507

**Published:** 2021-03-05

**Authors:** Jing Guo, Jiaxiao Wang, Keiko Iino, Masaru Tomita, Tomoyoshi Soga

**Affiliations:** ^1^Institute for Advanced Biosciences, Keio University, 246-2 Mizukami, Kakuganji, Tsuruoka 997-0052, Japan; ^2^Graduate School of Media and Governance, Keio University, 5322 Endo, Fujisawa 252-0882, Japan; ^3^Traditional Chinese Medicine Department, Emergency General Hospital, 29 Xibahe Nanli, Chaoyang District, Beijing 100028, China; ^4^Faculty of Environment and Information Studies, Keio University, 5322 Endo, Fujisawa 252-0882, Japan

## Abstract

**Background:**

Based on the theory of traditional Chinese medicine, Chinese herbs possess four different medicinal properties: hot, warm, cold, and cool. These serve as a reference guide for these herbal medicines. However, the molecular mechanisms supporting their relevance remain unclear.

**Methods:**

We performed metabolomics based on capillary electrophoresis-time-of-flight mass spectrometry (CE-TOF/MS) and multivariate data analysis for the structural identification of compounds of cold- and hot-natured Chinese herbs.

**Results:**

To this end, 30 selected herbs were analyzed and a total of 416 metabolites were identified via CE-TOF/MS, of which 193 compounds were detected in most herbs. The observed profiles offered the potential to understand the mechanism of association between the compounds and nature of the Chinese herbs. Comparison of the similarity in terms of chemical and molecular structures and content revealed that hot-natured herbs contained more nucleotides. In contrast, principal component analysis revealed the presence of more amino acid compounds in cold-natured herbs.

**Conclusion:**

Comparing the structural similarities between the samples using the Tanimoto coefficient revealed that a general non-specific structure was observed between cold- and hot-natured herbs; however, the distribution of the molecular groups seemed to contribute more toward the energy properties.

## 1. Introduction

In recent years, traditional Chinese medicine (TCM) has gained recognition worldwide as a valuable treatment for chronic and complex diseases. The World Health Organization has estimated that over 80% of the world's population relies on herbal medicines [1]. With centuries of clinical practice and development, recent endeavors have focused on scientific studies to establish the principles of TCM. However, ancient books on TCM generally present experience-based principles, obscuring the details needed for diagnosing and prescribing proper herbs for disease treatment. Therefore, it is important to clearly understand the nature of herbal medicines and illustrate their therapeutic uses in detail so as to provide a comprehensive guide to TCM inheritance because it is a treasure house for humans.

TCM recipes use herbs alone or in a mixture. Based on a patient's condition, a recipe is written for effective treatment using adjusted amounts or a combination of various ingredients by adding or deleting individual herbs. According to the therapeutic properties described in ancient books such as Shennong Ben Cao Jing and Compendium of Bencao, Chinese herbs represent complementary forces of “four natures and five flavors.” For rebalancing the Yin and Yang of the body, Chinese herbs can be classified into four groups: warm, hot, cold, and cool. These are the so-called four natures, also known as the four properties. In clinical application, the principle of healing in TCM is to balance the disturbed Yin–Yang in the body using appropriate therapy to restore the harmony of the entire body. To this end, hot-natured herbs are used for treating cold syndromes, and cold-natured herbs are used for treating heat syndromes. Although the four natures of Chinese herbs have been widely known by our ancestors for more than 2,000 years, the scientific basis of their classification remains unclear [2].

Among the TCM substances (botanical, animal, and mineral), more than 90% are of botanical origin, and the roots, stems, flowers, and fruits are medicinally useful [3]. Recent studies have proven the effects of the combination of herbal medicines [4–6]. Recently, coupled with statistical analysis, cold- and hot-natured Chinese herbs were investigated by using the state-of-the-art analytical techniques for the determination of amino acids and lipids [7]. The application of metabolomics analysis and multivariate data analysis to herbal medicine is also gaining increased attention [8–11]. Gas chromatography-mass spectrometry (GC-MS), liquid chromatography-MS (LC-MS), and nuclear magnetic resonance are used for the analysis of TCMs [12, 13].

Capillary electrophoresis-time-of-flight mass spectrometry (CE-TOF/MS) is an analytical technique used to qualitatively and quantitatively detect small-molecule compounds, and it is particularly well suited for the detection of polar and charged compounds in organisms [14, 15]. This technique has been broadly applied in biological samples, such as plants, and herbal preparations [9, 16]. When analyzing herbal species, providing a comprehensive evaluation for all metabolites remains a technical challenge [17].

In this study, 30 representative cold- and hot-natured herbs were selected for comparative analysis of the contents of charged small molecules in metabolites. Our study focused on the active ingredients, including alkaloids, organic acids, amino acids, flavonoids, and small-molecule polyphenols, that act on the human body [18]. Based on the identification results, the hot and cold properties of the compounds in Chinese herbs were characterized. These results may help elucidate the holistic molecular mechanisms underlying the determination of the intrinsic hot or cold nature of Chinese herbs.

## 2. Materials and Methods

### 2.1. Herbal Medicine Preparation

A total of 30 Chinese herbs were selected (Table 1), of which 15 were cold-natured and 15 were hot-nature herbs according to the description of the Chinese Pharmacopoeia (V. 2015). These herbs were purchased from Beijing Tong Ren Tang Group Co. Ltd. (Beijing, China). Researchers have analyzed the relationship between the four natures of herbal medicine and plant families [19]. However, the selection of herbs in our study was only based on only the four natures rather than on the plant family. Plant families with both hot/warm and cold properties, including Rutaceae, Asteraceae, and Ranunculaceae, were also present in the 30 selected herbs.

The preparation of herbs has been described in a previous study [16]. Briefly, 100 mg of each sample was frozen in liquid nitrogen and then homogenized into a single powder using a shocker (TOMY MS-100R) at 1,500 rpm for 180 × 2 s. After the preliminary treatment, the samples were processed by adding 1,250 *μL* of 60% ethanol solution containing 8 *μM* internal standard 1 (containing l-methionine sulfone, 2-morpholinoethanesulfonic acid, and d-camphor-10-sulfonic acid) and extracting the samples for 2 h. The supernatants were collected via centrifugation to obtain the extracted ingredients. The pellets containing plant residues, polysaccharides, and proteins were removed. The supernatants were transferred into 500-Da centrifugal filter tubes and recentrifuged at 9,100 × *g* and at 4°C for 3 h, followed by filtering the samples. After filtration, the liquid samples were placed in a vacuum dryer (35°C, 3 h) for drying. The dried samples were forcefully dissolved with Milli-Q water containing 200 *μ*M internal standard 2 (containing 3-aminopyrrolidine and trimesate) by vortexing.

### 2.2. Measurement Conditions for CE-TOF/MS

The instrumentation and measurement conditions used for CE-TOF/MS were as described elsewhere [15, 20]. In the positive ion mode, samples were separated in a fused silica capillary (50 *μm* i.d. × 100 cm) using 1 M formic acid as the electrophoretic and equilibrium buffer. Samples were injected under a pressure of 50 mbar for 3 s (approximately 3 *μL*) at an applied voltage of +30 kV. During the analysis, 50% (v/v) methanol containing 5 mM ammonium acetate and 0.5 M reserpine was used as the sheath liquid at a flow rate of 10 *μL*/min. In the negative ion mode, separation was performed on a capillary SMILE (+) (Nacalai Tesque, Kyoto, Japan) using 50 mM ammonium acetate (pH 8.5) as the mobile phase. Samples were injected under a pressure of 50 mbar for 30 s at an applied voltage of −30 kV.

### 2.3. Data Processing

The data measured via CE-TOF/MS were preprocessed using the MasterHands ver.2 software [21]. Noise-filtering, baseline correction, peak detection, and peak area integration were performed on mass/charge ratio (m/*z*) 0.02-width sliced electropherograms. The migration time was normalized using dynamic programming and simplex optimization. Peaks with small differences in their m/*z* values (<20 ppm) and normalized migration times (<1.0 min) were treated as features. External standards based on m/*z* values and migration times were used for identification and concentration calibration. To determine the concentrations of the compounds, the integral value (area) of the peak area, area of the sample compound, and area of the relative internal and external standards were calculated.

### 2.4. Statistical Analysis

#### 2.4.1. Discriminant Analysis

The concentration of each metabolite was represented as the average of three samples. The differences in metabolites between the two independent groups, i.e., cold- and hot-natured groups, were detected using the Mann–Whitney *U* test using the MeV TM4 software (Dana-Farber *Cancer* Institute, Boston, MA) [22].

#### 2.4.2. Principal Component Analysis (PCA)

PCA as an exploratory tool for data analysis used variables from a variety of components as a set of summary indices to observe the changing trend of all data. Analysis was conducted using the statistical software JMP ver.10 (SAS Institute Inc., Cary, NC, USA).

#### 2.4.3. Molecular Fingerprints and Similarity Searching

The commonly used algorithm to calculate the similarity of the compounds is the Tanimoto coefficient. Depending on the source of the structural information of the molecule provided by the public database of PubChem Compound, the structure data files of the identified metabolites were used for analysis [23]. The Tanimoto coefficient is an index to calculate the degree of similarity of two clusters using similarity calculation (equation (1)) by comparing their molecular fingerprints in chemical systems [24]. In equation (1), A and B are represented by vectors calculated from the local structures of various substances. When vectors A and B are exactly the same, the value to 1, it is not at all equal, the value to 0; for this reason, the closer to 1, the higher the similarity. The software Open Babel 2.3.0 was used to calculate the Tanimoto coefficients of all molecules [25].

(1) $$\begin{array}{c} T\left(A,B\right)=\frac{\left|A\cap B\right|}{\left|A\right|+\left|B\right|-\left|A\cap B\right|}. \end{array}$$

#### 2.4.4. Molecule Cluster Analysis

Molecule cluster analysis is a hierarchical clustering approach to discover the relationship between data by calculating the distance between the compounds extracted from the herbs and molecule cluster. In each of the succeeding steps, the closest clusters were merged to obtain a hierarchical structure. The Tanimoto coefficient was calculated based on the similarity between the substances. Molecule cluster analysis was performed using the Mev TM4 V4.6 analysis software [26] based on Spearman correlation coefficient (nonparametric method) and was classified based on average index.

## 3. Results

### 3.1. Profiling of the Charged Metabolites in TCMs

**Figure 1:**
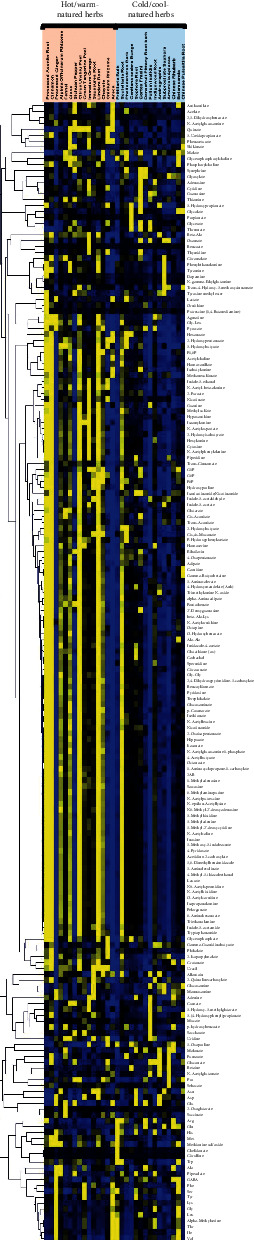
The heatmap is based on the 193 identified compounds from 30 extracts of Chinese herbs. Rows represent compounds and columns represent the Chinese herb samples. The orange box groups the 15 hot/warm-natured herbs and the blue box groups the 15 cold/cool-natured herbs. Color key indicates the relative concentrations of the identified compounds: yellow indicates higher concentrations, black indicates medium concentrations, and blue indicates lower concentrations. Each herb had three duplicate samples.

CE-TOF/MS-based metabolomics analysis was performed to detect the metabolites, including amino acids, organic acids, alkaloids, and nucleotides, in Chinese herbs. In total, 416 charged small molecules matched with the compounds in our standard library. Notably, despite a clear difference in metabolites between different herbs, there was a general metabolite-by-metabolite similarity among the herbs, and 193 identical metabolites were detected in most herbs. In the present study, to accelerate the similarity search, the lead 193 metabolites identified from over 16 herbs were screened using metabolomics for cluster analysis. The heatmap clusters based on the metabolite profiles were generated using the dataset to clarify the distribution of the identified metabolites between different groups.  Figure 1 shows that the metabolites concentrated in the middle of the heatmap tended to be present in high concentrations in hot-natured herbs and in low concentrations in cold-natured herbs. However, few herbs had lower similarities than other herbs, for example, Rhizoma Zingiberis, Pericarpium Zanthoxyli, Rhizoma Chuanxiong, and Semen Arecae in hot-natured herbs and Radix Scutellariae in cold-natured herbs.

**Figure 2:**
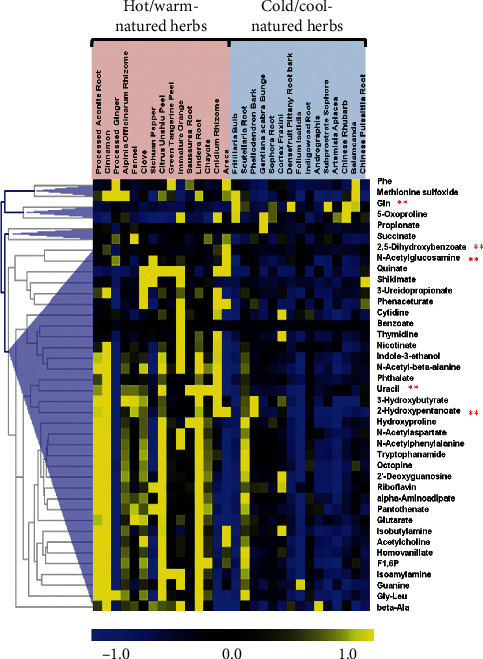
Heatmap cluster analysis of the metabolites with significant differences (*p* < 0.05) between the varied groups of hot- and cold-natured herbs. Rows represent metabolites and columns represent herbs. The orange box groups the 15 hot/warm-natured herbs and the blue box groups the 15 cold/cool-natured herbs. Color key indicates the relative concentrations of the identified compounds: yellow indicates higher concentrations, black indicates medium concentrations, and blue indicates lower concentrations. In addition, the *p* values highlighted with two stars are less than 0.01. PCA of the charged metabolites in TCMs.

Figure 2 shows the cluster analysis results of the significant differences in metabolite concentrations between hot/warm- and cold/cool-natured herbs. From the 193 metabolites identified in each herb in Figure 1, more than 40 metabolites with significant differences (*p* < 0.05) in concentrations between hot/warm- and cold/cool-natured herbs were selected via cluster analysis. In particular, the concentrations of 2,5-dihydroxybenzoate, 2-hydroxypentanoate, n-acetylglucosamine, and uracil were significantly higher in hot/warm-natured herbs than in cold-natured herbs (*p* < 0.01). In contrast, glutamine concentration was significantly higher in cold-natured herbs than in hot/warm-natured herbs (*p* < 0.01) (Figure 2).

**Figure 3:**
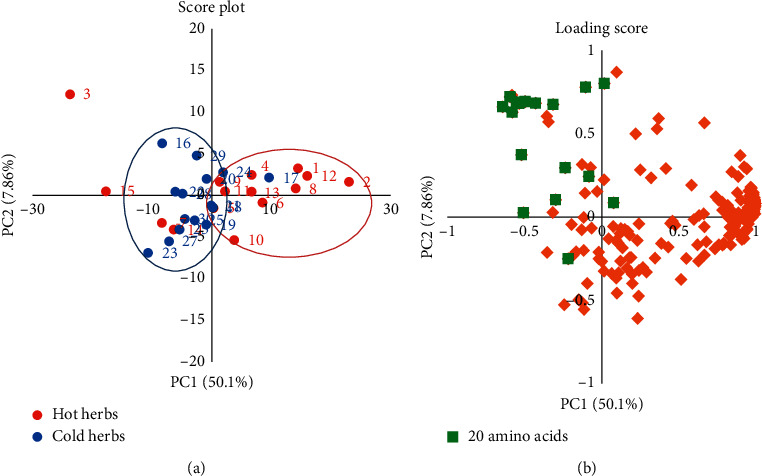
Principal component analysis score plots (a) and loading scores (b) (PC1 vs. PC2) of the Chinese herbs. The *x*-axis is principal component 1 (PC1), and the *y*-axis is principal component 2 (PC2). PC1 and PC2 describe around 50.1% and 7.86% of the total variability, respectively. In the score plot, red represents hot/warm-natured herbs (hot herbs), and blue represents cold/cool-natured herbs (cold herbs). Furthermore, the numbers correspond to the names of the herbs listed in Table 1. In the loading score, the green squares represent the 20 basic amino acids, and the orange diamonds represent all other metabolites.

As a decomposition approach, PCA allowed the original metabolite data to be reduced to a few principal components of the data to obtain more detailed metabolic variations among the herbs. The PCA score plots (blue for cold/cool-natured herbs and red for hot/warm-natured herbs) and loading plots of the 30 Chinese herbs are presented in Figure 3. The first two principal components accounted for 58% of the overall variability. PC1 scores (50.1%) revealed that the spectra of hot/warm-natured and cold/cool-natured herbs were separate, except for that of individual herbs (Rhizoma Zingiberis and Semen Arecae). Overall, except for some deviating data points, the metabolite scores of the hot/warm-natured herbs drifted toward the positive axis, whereas the scores of the cold/cool-natured herbs were focused on the negative axis. Meanwhile, the loading score plots showed that 20 kinds of amino acids contributed to the negative axis, agreeing with the score plots of cold/cool-natured herbs. It seemed that there were respective differences between hot/warm-natured and cold/cool-nature herbs. As indicated by the results, the amino acid concentration was in fact closely related to cold/cool-natured herbs.

### 3.2. Molecular Similarity Analysis

**Figure 4:**
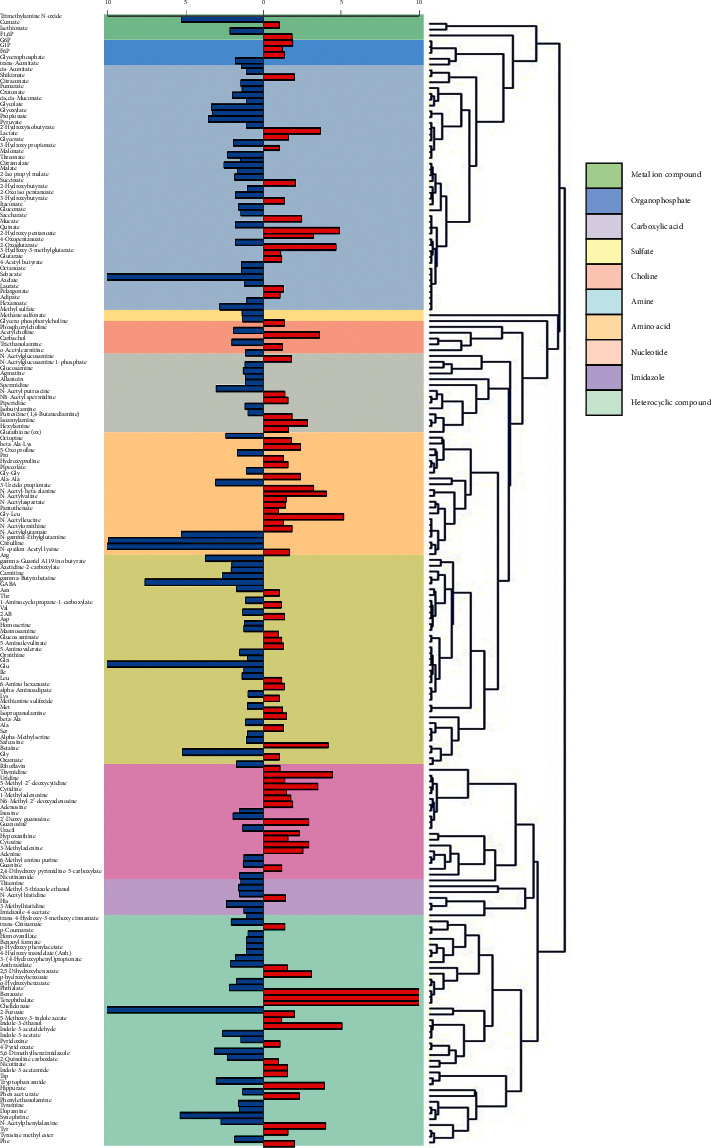
Molecular structure similarity of the metabolites and the metabolite content ratio of cold-to hot-natured herbs. The hierarchical tree is constructed using the results of Tanimoto coefficients, and the similarity approach between the molecules of various metabolites is distance-based. The common structure of each classification is represented on the right side of the hierarchical tree. For easy comparison of the ratio of cold-to hot-natured herbs, the left vertical bar chart displays these ratios calculated from the average values of each metabolite. The red-colored bars indicate the high metabolite concentration in hot/warm-natured herbs, and the blue-colored bars indicate the high metabolite concentration in cold-natured herbs.

Because most of the compounds were derived from a common skeleton building block in the plant metabolic network, the structural similarity of compounds might play an important role in identifying the herb as hot- or cold-natured. Therefore, a hierarchical tree constructed using cluster analysis was investigated to support compound information on relationships with the properties of Chinese herbs. Using the Tanimoto coefficient, the extracted information of the selected 193 metabolites from PubChem Compound was used to calculate the structural similarity between the samples. The samples were arranged into a hierarchy by grouping the common structure of the metabolites (Figure 4). In addition, after assigning the major metabolites, the relative concentrations of these metabolites were measured to calculate the mean values of hot- and cold-natured herbs. By comparing with mean values, the relative ratios of cold- to hot-natured herbs were calculated to observe the contributions to each group. The results showed that although a non-specific structure was generally observed between cold- and hot-natured herbs, the nucleotides in the red box accounted for a large proportion in most hot-natured herbs.

**Figure 5:**
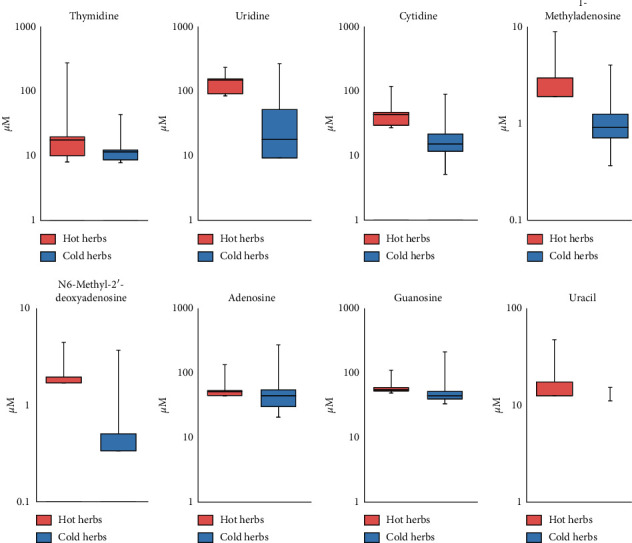
Nucleoside metabolites are more widely distributed in hot-natured herbs than in cold-natured herbs. Boxplot shows the concentration of each metabolite in hot- and cold-natured herbs. The pink box represents the 15 hot-natured herbs (hot herbs), and the blue box represents the 15 cold-natured herbs (cold herbs). Boxplots describe the upper quartile (Q3), median, and the lower quartile (Q1) values. Upper and lower limit whiskers describe the maximum and minimum values in the data.

Next, we visualized the distribution of nucleosides and related concentration of compounds in both hot- and cold-natured herbs using a boxplot (Figure 5). Thymidine, uridine, cytidine, 1-methyladenosine, N6-methyl-2′-deoxyadenosine, adenosine, guanosine, and uracil were slightly more distributed in hot-natured herbs (pink) than in cold-natured herbs (blue). Soluble nucleotides and analogs have been reported to be the most important bioactive ingredients in some TCMs [27].

## 4. Discussion

The four-natured principles serve as the reference guide for TCM recipes. However, till date, the underlying mechanism of such different properties on therapeutic effects has not been fully identified at molecular level as a complex research system. This is generally believed to be related to the large number of molecular groups in Chinese herbs. In this study, 15 hot/warm-natured herbs and 15 cold-natured herbs were selected by analyzing their ionic metabolites via CE-TOF/MS detection, followed by multiple statistical analysis approaches to investigate the correlation of properties between each molecular group. The approaches included PCA, discriminant analysis, Tanimoto coefficient analysis, and molecular cluster analysis. In total, 416 metabolites from 30 Chinese herbs were recognized, and it was noted that 193 of the 416 compounds were found in more than 16 herbs. The CE-TOF/MS-based metabolomics with multivariate data analysis enabled the identification of compounds between cold- and hot-natured herbs. Although the levels of compounds, including amino acids, nucleosides, and nucleotides, varied in both cold- and hot-natured herbs due to individual differences, the results via metabolomics PCA, Mann–Whitney *U* test, and structural similarity analysis still illustrated some regular characteristics among the different groups. Metabolomics analysis of the 193 metabolites indicated significant differences in the contents of the 40 metabolites between hot- and cold-natured herbs (Figure 2). In contrast, more amino acid compounds were observed in cold-natured herbs via PCA (Figure 3). Comparative analysis of the molecular similarity of the chemical and molecular structures and contents revealed that hot-natured herbs had more number of nucleotides (Figure 4).

Many studies have focused on antioxidant activities in cellular or mouse experimental systems [28, 29]. However, more quantitative studies on the four natures of Chinese herbs have focused on microelement and chemical compositions [30]. While discussing the effects of chemical compositions on the cold/hot natures of Chinese herbs, the theory of generalized oxidation and reduction was hypothesized to be associated with these kinds of thermal properties [31]. In general, metabolism of the human body can be broadly categorized into catabolism and anabolism [32]. In sharp contrast, cold-natured herbs consist of more amino acid compounds, which are the basic building blocks for the anabolic processes of cellular growth or function (Figure 3). The absorption of amino acids is mainly used for protein synthesis, requiring energy in the form of ATP. Energy consumption leads to a cold effect in nature. Therefore, cold- and hot-natured herbs can be distinguished from the viewpoint of metabolism. In addition, it has been shown that warm/hot-natured herbs contain more nucleotides and their derivatives (Figures 4 and 5). Immunity, inflammation, and cancer can all be regulated by extracellular nucleosides [33–35]. Furthermore, based on the functional activities of the metabolites, Liang et al. searched the protein targets of active compounds in the PubChem database and found that immune regulation was more related to hot-natured herbs and that cold-natured herbs possess the tendency to impact cell growth and proliferation [36]. Therefore, the high concentration of nucleosides and analogs in hot-natured herbs may play a role in immune regulation.

Although each herb has different actions and applications, metabolomics analysis indicates that each herb contains the common biologically active ingredients, such as amino acids, organic acids, and nucleoside or nucleotide compounds. Therefore, it should be noted that the different energy natures of hot, warm, cold, and cool are not merely determined by one or a certain class of chemical molecules but by the combined effects of all of ingredients, the so-called molecular groups. Warm/hot-natured herbs are more prevalent in those molecular groups prone to oxidation reactions, such as nucleotides. On the other hand, cold/cool-natured herbs have the predominant components prone to reduction reactions, such as amino acids.

## 5. Conclusions

In this study, to determine the key compounds in Chinese herbs of the four inherent energy properties, CE-TOF/MS-based metabolomics with statistical analysis was performed to measure the metabolites of the 30 selected Chinese herbs. The results showed that organic acids and nucleotide compounds were accumulated in warm/hot-natured herbs, whereas basic amino acids such as glutamine and nucleoside compounds were present in higher concentrations in cold-natured herbs. PCA revealed that there are important differences between warm/hot- and cold/cool-natured herbs and that the molecular groups and energy properties of herbs are closely related to each other. Overall, these results show that our CE-TOF/MS-based metabolomics approach provides a powerful tool to assess the relationship between the molecular mechanisms underlying the four natures of Chinese herbs. This approach will help establish the theoretical basis of TCM or traditional medicine.

## Figures and Tables

**Table 1:** Names and properties of the 30 Chinese herbs used for metabolite extraction.

Number	Name	Latin index	Family	Medicinal part	Natures	Flavor
1	Radix Aconiti Praeparata	*Aconitum carmichaelii* Debeaux	Ranunculaceae	Root	Hot	Pungent, sweet
2	Cortex Cinnamomi	*Cinnamomum cassia* Nees ex Blume	Lauraceae	Bark	Hot	Pungent, sweet
3	Rhizoma Zingiberis	*Zingiber officinale* Rosc.	Zingiberaceae	Rhizome	Hot	Pungent
4	Rhizoma Alpiniae Officinarum	*Alpinia officinarum* Hance	Zingiberaceae	Rhizome	Hot	Pungent
5	Fructus Foeniculi	*Foeniculum vulgare* mill.	Umbelliferae	Fruit	Warm	Pungent
6	Flos Caryophylli	*Syzygium aromaticum* (*L.*) Merr. and L. M. Perry	Myrtaceae	Flower bud	Warm	Pungent
7	Pericarpium Zanthoxyli	*Zanthoxylum bungeanum* Maxim.	Rutaceae	Pericarp	Warm	Pungent
8	Pericarpium Citri Reticulatae	*Citrus reticulata* Blanco	Rutaceae	Pericarp	Warm	Pungent, bitter
9	Pericarpium Citri Reticulatae Viride	*Citrus reticulata* Blanco	Rutaceae	Pericarp	Warm	Pungent, bitter
10	Fructus Aurantii Immaturus	*Citrus* × *aurantium* Linn.	Rutaceae	Fruit	Warm	Pungent, bitter
11	Radix Aucklandiae	*Aucklandia lappa* DC.	Asteraceae	Root	Warm	Pungent, bitter
12	Radix Linderae	*Lindera aggregata* (*Sims*) Kosterm.	Lauraceae	Root	Warm	Pungent
13	Fructus Citri Sarcodactylis	*Citrus medica* var. *Sarcodactylis* (*Noot.*) Swingle	Rutaceae	Fruit	Warm	Pungent, bitter
14	Rhizoma Chuanxiong	*Ligusticum sinense* “*Chuanxiong*” S. H. Qiu et al.	Umbelliferae	Rhizome	Warm	Pungent
15	Semen Arecae	*Areca catechu* Linn.	Palmae	Seed	Warm	Pungent, bitter
16	Bulbus Fritillariae Cirrhosae	*Fritillaria cirrhosa D.* Don	Liliaceae	Rhizome	Cold	Bitter, sweet
17	Radix Scutellariae	*Scutellaria baicalensis* Georgi	Labiatae	Root	Cold	Bitter
18	Cortex Phellodendri	*Phellodendron chinense* Schneid.	Rutaceae	Bark	Cold	Bitter
19	Radix Gentianae	*Gentiana scabra* Bunge	Gentianaceae	Rhizome	Cold	Bitter
20	Radix Sophorae Flavescentis	*Sophora flavescens Ait.*	Papilionaceae	Root	Cold	Bitter
21	Cortex Fraxini	*Fraxinus chinensis* subsp. *Rhynchophylla (Hance) E.* Murray	Oleaceae	Bark	Cold	Bitter
22	Cortex Dictamni	*Dictamnus dasycarpus* Turcz.	Rutaceae	Velamen	Cold	Bitter
23	Folium Isatidis	*Isatis tinctoria* Linn.	Cruciferae	Leaf	Cold	Bitter
24	Radix Isatidis	*Isatis tinctoria* Linn.	Cruciferae	Root	Cold	Bitter
25	Herba Andrographis	*Andrographis paniculata (Burm. f.)* Nees	Acanthaceae	Stem leaf	Cold	Bitter
26	Radix Sophorae Tonkinensis	*Euchresta japonica* Regel	Papilionaceae	Rhizome	Cold	Bitter
27	Herba Artemisiae Annuae	*Artemisia annua L*.	Compositae	Stem leaf	Cold	Bitter, pungent
28	Radix et Rhizoma Rhei	*Rheum palmatum* Linn	Polygonaceae	Rhizome	Cold	Bitter
29	Rhizoma Belamcandae	*Belamcanda chinensis (Linn.)* DC.	Iridaceae	Rhizome	Cold	Bitter
30	Radix Pulsatillae	*Pulsatilla chinensis (Bunge)* Regel	Ranunculaceae	Root	Cold	Bitter

## Data Availability

The data used to support the findings of this research are available from the corresponding author upon request.
